# Preoperative Hemoglobin Threshold as a Predictor of Transfusion Risk in Pregnant Patients: An Observational Study for Informing Patient Blood Management Strategies in a Tertiary Care Facility in Romania

**DOI:** 10.3390/medicina62061079

**Published:** 2026-06-02

**Authors:** Mirela Andreea Marcu, Ancuța Iacob, Carmen Lidia Chițescu, Mihaela Roxana Olita, Dana Rodica Tomescu

**Affiliations:** 1 Carol Davila University of Medicine and Pharmacy, 050474 Bucharest, Romania; mirela-andreea.cotet@drd.umfcd.ro (M.A.M.); dana.tomescu@umfcd.ro (D.R.T.); 2 Department of Obstetrics and Gynecology, Emergency Country Clinical Hospital of Braila, 810325 Braila, Romania; 3 Research Centre in the Medical-Pharmaceutical Field, Department of Pharmaceutical Science, Faculty of Medicine and Pharmacy, “Dunarea de Jos” University of Galati, 800008 Galati, Romania; ancuta.dinu@ugal.ro (A.I.); carmen.chitescu@ugal.ro (C.L.C.); 4 Anesthesiology and Intensive Care, Fundeni Clinical Institute, 022328 Bucharest, Romania

**Keywords:** anaemia, pregnancy, patient blood management, transfusion risk, hemoglobin

## Abstract

*Background and Objectives:* Preoperative anaemia represents a key modifiable risk factor in obstetrics. Within the framework of Patient Blood Management (PBM), establishing precise hemoglobin (Hb) thresholds is essential for optimal clinical decision-making. This study aimed to assess the predictive value of preoperative hemoglobin levels and to determine the optimal cutoff associated with transfusion risk. *Materials and Methods:* A retrospective analysis was performed on 932 pregnant women. The association between preoperative hemoglobin, anticoagulant therapy, mode of delivery and maternal age with the need for red blood cell transfusion was evaluated using binary logistic regression and Receiver Operating Characteristic (ROC) curve analysis with the Youden index. *Results:* Red blood cell transfusion was required in 5.2% (n = 48) of the study population. Logistic regression identified preoperative hemoglobin as the strongest independent predictor (*p* < 0.001, OR = 0.216, 95% CI: 0.153–0.306), indicating that each 1 g/dL increase in Hb reduced the likelihood of transfusion by 78.4%. Anticoagulant therapy and age were not significant independent predictors (*p* > 0.05). ROC analysis demonstrated excellent predictive performance, with an Area Under the Curve (AUC) of 0.875 (95% CI: 0.823–0.927, *p* < 0.001). The optimal threshold for predicting transfusion risk was 10.9 g/dL (sensitivity: 89.6%, specificity: 60.5%). *Conclusions:* Preoperative hemoglobin concentration is the primary determinant of transfusion risk, outweighing the influence of clinical comorbidities. The integration of PBM protocols designed to sustain hemoglobin levels above 10.9 g/dL is essential to reduce perioperative transfusion requirements and to promote improved maternal safety and clinical outcomes.

## 1. Introduction

According to the World Health Organization (WHO), anaemia is an important global public health problem, particularly among pregnant women [1,2]. The WHO estimates that approximately 35.5% of pregnant women worldwide are affected by anaemia, with lower prevalence in Europe, where the rate is around 25% [3]. Pregnancy may be regarded as a physiological “stress test” that induces complex hemodynamic changes, characterized by plasma volume expansion exceeding the increase in red cell mass [4]. This physiological dilutional anaemia, together with the increased iron requirements of the feto-placental unit, rapidly depletes maternal ferritin stores [5,6]. Thus, this condition represents not only a nutritional deficiency, but also a consequence of the imbalance between the increased metabolic demands of the fetus and the mother’s limited hematologic adaptative capacity [7,8]. Such aspects lead to an increased risk of perioperative complications, making preoperative hemoglobin optimization a clinical necessity rather than a therapeutic option [9,10]. In the second and third trimesters, daily iron requirements may increase up to tenfold, turning pregnancy into a state that challenges hematologic homeostasis [11]. When iron stores are already low at the beginning of pregnancy, the patient may enter a vicious cycle of functional deficiency that culminates in preoperative anaemia at the time of delivery [12]. The importance of this global context lies in the fact that a patient who enters the operating room (for caesarean section) or the delivery room with an already reduced hemoglobin level has a limited safety reserve. In this context, even blood loss considered physiological (approximately 500–1000 mL) may trigger the need for red blood cell transfusion [13].

In this scenario, preoperative anaemia may be a predictor of maternal morbidity, in the sense that it can destabilize the entire perioperative recovery process [14]. Anaemic pregnant patients have a significantly increased risk of postpartum hemorrhage leading to uterine atony [15]. Moreover, anaemia impairs immune response and collagen synthesis, increasing the incidence of surgical site infections and prolonging hospital stay, which generates additional costs for the healthcare system [7]. In current practice, red blood cell transfusion is often used as a last-resort intervention to correct intraoperative blood loss or severe anaemia [16]. However, the modern literature emphasizes that transfusion itself is an independent risk factor for immunological and inflammatory reactions, logistical errors, and the use of limited resources [17,18]. Although these risks are well recognized, the major gap in the literature is the absence of an early warning threshold. Clinicians tend to intervene when hemoglobin levels falls below 7–8 g/dL, yet our data suggest that risk begins to rise much earlier. Without a robust predictive model, the opportunity for intravenous iron therapy or red blood cell optimization may be lost.

**Figure 1:**
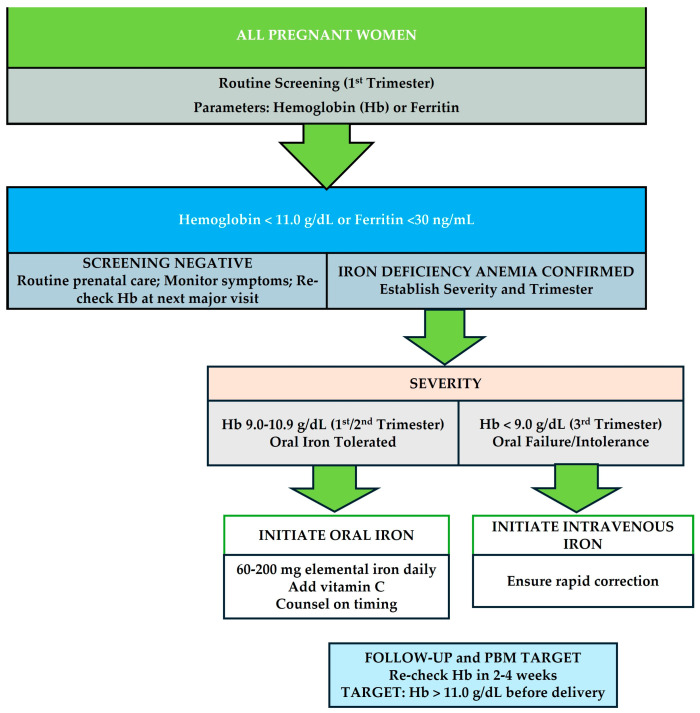
Algorithm for the Management of Iron Deficiency Anaemia (IDA) in Pregnancy (Patient Blood Management Approach) (Adapted from [22]).

Most modern concepts regarding the management of preoperative anaemia in obstetrics are based on patient blood management (PBM) strategies (Figure 1), which aim to maintain hemoglobin concentration, optimize hemostasis, minimize blood loss and limit red blood cell transfusion during delivery, in order to improve maternal and fetal outcomes [19].

Unlike the hemoglobin threshold of 12 g/dL in non-pregnant women, the WHO defines anaemia in pregnant women as hemoglobin < 11 g/dL, regardless of gestational age [20]. However, the Centers for Disease Control and Prevention (CDC) define anaemia in pregnant women as hemoglobin < 11 g/dL in the first and third trimesters and hemoglobin < 10.5 g/dL in the second [21].

The implementation of Patient Blood Management (PBM) programs has become a cornerstone of modern surgical practice, and it is endorsed by the WHO as a fundamental patient safety strategy. In obstetrics, the application of Pillar I of PBM, namely the preoperative optimization of red cell mass, is particularly important. This strategy reframes the management of anaemia from a merely numerical correction into a clinically meaningful intervention aimed at reducing maternal morbidity [23]. The present study highlights that the effectiveness of Pillar I critically depends on the identification of a precise predictive hemoglobin threshold, which would enable timely clinical intervention prior to delivery, thereby preserving limited blood resources and minimizing the immunological risks associated with transfusion.

Although Patient Blood Management (PBM) programs are internationally recognized, their implementation in obstetrics units in Romania requires robust local data that reflect the characteristics of the studied population and current clinical practice. The primary objective of the study was to evaluate the predictive value of preoperative hemoglobin for the need for red blood cell transfusion and to establish an optimal cut-off point to guide PBM interventions in obstetrics patients in Romania. In parallel, we sought to demonstrate that hemoglobin represents the principal determinant of transfusion risk, independent of the other potentially cofounding clinical factors, such as age or anticoagulant therapy. The secondary objective was to identify, through ROC curve analysis, a critical hemoglobin threshold that could serve as an objective marker for initiating preoperative hematologic optimization protocols. The study was based on the hypothesis that a preoperative hemoglobin level below 11 g/dL is associated with a significantly increased likelihood of transfusion, regardless of anticoagulant therapy.

## 2. Materials and Methods

### 2.1. Study Design and Setting

We conducted a retrospective, observational, non-randomized, analytical study covering the period between 2023 and 2024. The study was performed within the 2nd Level Obstetrics and Gynecology Department of the Emergency County Clinical Hospital of Brăila, Romania. The research protocol was reviewed and approved by the Hospital’s Ethics Committee, ensuring compliance with ethical standards for retrospective data collection.

### 2.2. Patient Selection and Eligibility

The study population consisted of a final sample of 932 pregnant women, aged between 18 and 40 years, who were admitted for delivery or associated obstetric care at the Emergency County Clinical Hospital of Brăila during the 2023–2024 period. Eligibility was strictly defined by the availability of complete medical records, including documented pre- and postoperative Hb levels, delivery mode, and specific clinical data regarding anaemia, thrombophilia, or anticoagulant therapy. In this study, anemia was defined as a preoperative Hb level below 11 g/dL, in accordance with the WHO guidelines for pregnant women [24]. The severity of anemia was classified as mild (10.0–10.9 g/dL), moderate (7.0–9.9 g/dL), and severe (<7.0 g/dL) [25]. The timing of Hb measurement corresponded to the blood tests performed at the time of admission for delivery.

To ensure a homogenous and clinically relevant cohort, we excluded patients outside the specified age range, those with incomplete clinical data, and individuals with chronic hematological disorders unrelated to pregnancy or non-iron deficiency anaemias, such as Vitamin B12/folate deficiency, hemolytic anaemia, or anaemia associated with chronic systemic diseases. This rigorous selection process ensured that the analysis focused on the predictive value of hemoglobin within a standard obstetric population, minimizing potential confounding factors from pre-existing hematological pathologies.

### 2.3. Data Collection and Variables

Data were manually extracted from the patients’ medical observation charts. Continuous variables (age, weight, height, pre- and post-operative hemoglobin levels, platelet count, hematocrit, and MCV) were initially analyzed using descriptive statistics, with the calculation of minimum and maximum values, mean and standard deviation, median and interquartile range, as well as measures of distribution shape (skewness and kurtosis). The distribution of each variable was assessed using graphical methods (histograms with superimposed normal curves, Q–Q and P–P plots), as well as formal tests of normality (Shapiro–Wilk and Anderson–Darling). Given the large sample size, the decision regarding the subsequent use of parametric or nonparametric tests was based on an evaluation of the normality tests, the shape indicators, and the graphical analysis of the distributions.

The primary dependent variable was the administration of red blood cell (RBC) transfusion, analyzed as both a categorical and binary outcome. The core independent variable was the preoperative Hb level, which was evaluated for its diagnostic accuracy and optimal cut-off point. To control for potential confounding factors, the statistical analysis also incorporated maternal age, delivery mode (vaginal delivery vs. Cesarean section), the documented presence of thrombophilia, and the perioperative use of anticoagulant therapy or aspirin. Out of the total sample, 342 women (36.7%) had a vaginal delivery, and 589 women (63.3%) underwent a cesarean section. These variables provided the basis for the Pearson Chi-Square comparisons, Binary Logistic Regression, and ROC curve analysis, ensuring a comprehensive evaluation of transfusion risk predictors.

### 2.4. Statistical Analysis

Statistical processing was performed using SPSS Statistics, version 22 and Microsoft Excel. Categorical variables were compared using the Pearson Chi-Square test. The decision for red blood cell transfusion was based on institutional guidelines (hemoglobin <7 g/dL with associated symptoms of anemia). To identify independent risk factors for blood transfusion, a Binary Logistic Regression model was employed; variables were entered into the model based on clinical significance and univariate screening (*p* < 0.1). The diagnostic accuracy of preoperative hemoglobin and Body Surface Area (BSA) was assessed using ROC curve analysis, with the optimal cut-off point determined via the Youden Index (J = Sensitivity + Specificity − 1). Cases with missing data were excluded from the multivariate analysis to ensure data consistency. A *p*-value of <0.05 was considered statistically significant.

## 3. Results

The analyses of the distribution of continuous variables revealed a heterogeneous pattern with regard to statistical normality, requiring a differentiated approach in the subsequent analytical steps. These variables were described by median (Table 1) and interquartile range and were subsequently analyzed using parametric and nonparametric statistical methods, according to the data distribution.

### 3.1. Demographic and Clinical Characteristics

**Figure 2:**
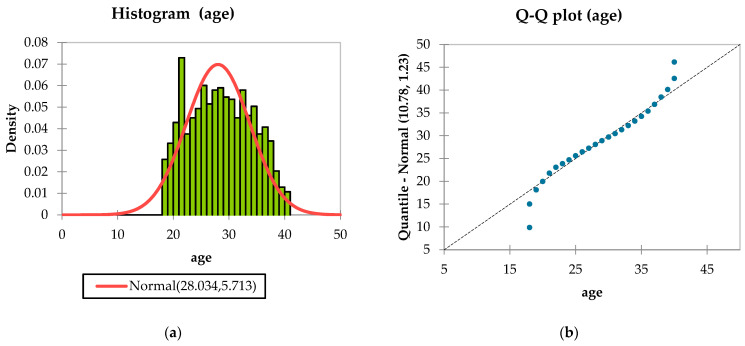
Histogram of patient age distribution (**a**), with a superimposed theoretical normal curve, and the corresponding Q–Q plot (**b**).

The patients’ age ranged from 18 to 40 years, with a mean of 28.03 ± 5.71 years. The distribution of continuous variables was assessed using the Shapiro–Wilk and Anderson–Darling tests, complemented by visual inspection of histograms and Q–Q and P–P plots (Figure 2a,b). Although the normality tests indicated statistically significant deviations (*p* < 0.001), graphical assessment and distribution indicators (skewness = 0.09; kurtosis = −0.99), suggested only a minimal departure from normality, with minor deviations at the distribution extremes.

**Figure 3:**
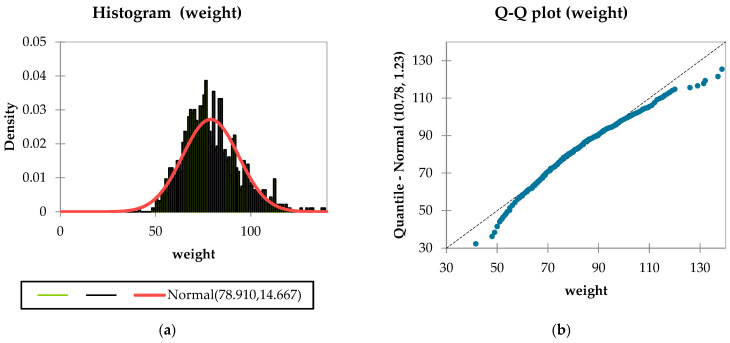
Histogram of patients’ body weight distribution (**a**), with a superimposed theoretical normal curve, and the corresponding Q–Q plot (**b**).

The patients’ body weight ranged between 41.6 and 138.6 kg, with a median of 77 kg (IQR 69.0–87.5). The distribution was right-skewed, with increased dispersion of values, reflecting substantial weight heterogeneity within the study population. The Anderson–Darling test showed a significant deviation from normality (A^2^ = 5.197; *p* < 0.001), a finding supported by visual assessment of histograms and Q–Q and P–P plots (Figure 3a,b). In addition, distributional indicators for body weight suggested positive skewness (skewness = 0.64) and a leptokurtic tendency (kurtosis = 0.60), consistent with a right-skewed distribution. As a variable with a skewed distribution, body weight was described using the median and interquartile range and subsequently analyzed using nonparametric statistical methods.

**Figure 4:**
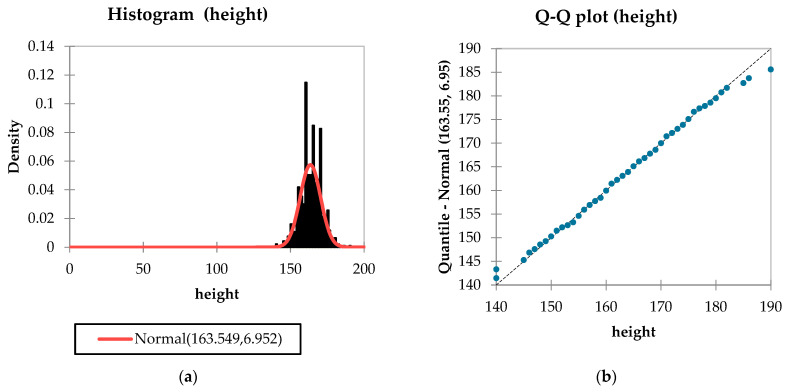
Histogram of patients’ height (**a**), with superimposed theoretical normal curve, and the corresponding Q–Q (**b**).

The distribution of patients’ height was unimodal and approximately symmetric. The median height was 164 (interquartile range: 160–168 cm), while the mean value was 163.5 ± 7.0 cm, with observed values ranging from 140 to 190 cm. The skewness coefficient was 0.004, a value very close to zero, indicating an essentially symmetric distribution within the analyzed sample. The kurtosis coefficient was 0.240, suggesting a distribution closely approximating normality in terms of both peakedness and tail behavior. Graphical assessment further supported the assumption that height could be treated as approximately normally distributed (Figure 4a,b). The histogram confirmed a unimodal pattern, with the majority of values clustered between 155 and 175 cm and a good agreement between the empirical distribution and the theoretical normal curve.

**Figure 5:**
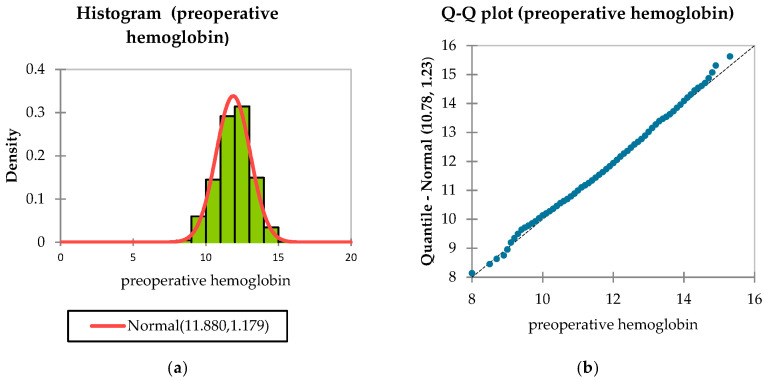
Histogram of preoperative hemoglobin values in the study cohort (**a**), with superimposed theoretical normal curve, and the corresponding Q–Q plot (**b**).

The preoperative hemoglobin values ranged from 8.0 g/dL to 15.3 g/dL, with a mean level of 11.88 g/dL. The variability of hemoglobin levels was moderate, as indicated by a standard deviation of 1.18 g/dL, suggesting a relatively low dispersion around the central value. Regarding measures of central tendency, the median was 11.90 g/dL (IQR 11.10–12.70 g/dL), resulting in an interquartile range of 1.6 g/dL. The close agreement between the mean and median indicates an overall approximately symmetric distribution of hemoglobin values. The Shapiro–Wilk normality test yielded a W value of 0.996, with a *p*-value of 0.009, while the Anderson–Darling test produced an A^2^ value of 1.265, and a *p*-value of 0.003, with both results suggesting a statistically significant deviation from normality (*p* < 0.05). However, these findings should be interpreted in the context of the large sample size, as normality tests are highly sensitive to even minor deviations when the number of observations is substantial. Analysis of the distribution shape using Pearson’s coefficients revealed a slightly negative skewness (−0.19), indicating a marginally longer tail toward lower hemoglobin values, and a kurtosis value close to (−0.10), suggesting an approximately mesokurtic distribution, comparable to the normal distribution. The histogram also demonstrated a unimodal, roughly symmetric pattern, with a good overlap with the theoretical normal curve. Likewise, the Q–Q plot showed an excellent alignment of data points along the reference line, with only minor deviations at the distribution tails (Figure 5a,b).

**Figure 6:**
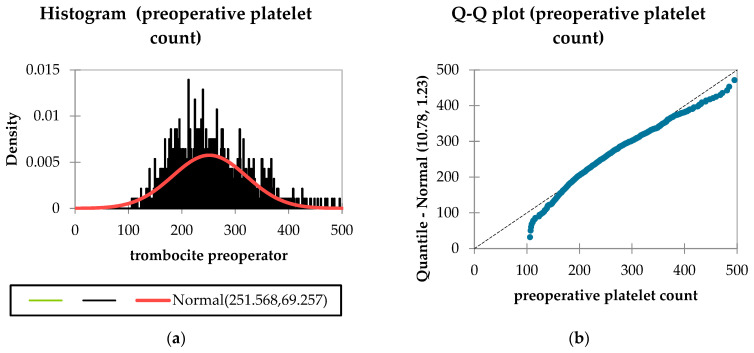
Histogram of preoperative platelet counts in the study cohort (**a**), with the superimposed theoretical normal distribution curve, and the corresponding Q–Q plot (**b**).

The preoperative platelet count exhibited considerable variability, with values ranging from 106 × 10^3^/µL to 495 × 10^3^/µL, indicating marked biological heterogeneity in platelet count within the study population. The mean platelet count was 251.6 × 10^3^/µL, with a standard deviation of 69.3 × 10^3^/µL, indicating relatively wide dispersion around the mean. The median was 244 × 10^3^/µL (IQR 200 × 10^3^–294 × 10^3^/µL), closely approximating the arithmetic mean; however, the interquartile range of 94 × 10^3^/µL confirms substantial variability among the central observations. The distribution of platelet counts demonstrated positive skewness (skewness = 0.576), suggesting a longer right tail attributable to the presence of elevated platelet values, as well as slight positive kurtosis (kurtosis = 0.143), consistent with a distribution broadly approximating normality but with a mild leptokurtic tendency. The histogram showed a right-skewed pattern, with the principal peak located in the 200–300 × 10^3^/µL range. In addition, the Q–Q plot revealed systematic deviations from the theoretical normal line, particularly at the lower and upper extremes of the distribution. Normality testing yielded a statistically significant result (*p* < 0.0001), leading to rejection of normality (Figure 6a,b), and supporting the subsequent use of nonparametric statistical methods.

**Figure 7:**
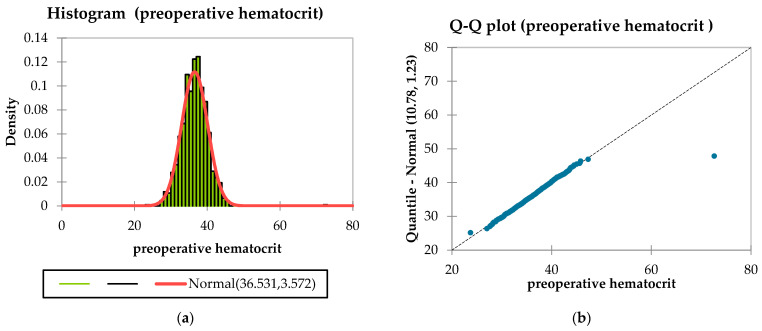
Histogram of preoperative hematocrit values in the study cohort (**a**), superimposed theoretical normal distribution curve, and the corresponding Q–Q plot (**b**).

The hematocrit values ranged from 23.7% to 72.6%, with a mean of 36.53%, and a standard deviation of 3.57%, indicating a moderate dispersion around the central value. The median was 36.6%, closely approximating the mean, which suggests an overall relatively symmetric distribution of the data. Assessment of distribution shape revealed moderate positive skewness (Skewness + 1.026), whereas the markedly elevated kurtosis value (Kurtosis = 10.616) indicated a leptokurtic distribution, characterized by a sharper peak and the presence of extreme observations. These findings were consistent with the graphical analysis, including the histogram and the Q–Q plot, which demonstrated a substantial concentration of values around the central tendency, as well as isolated observations, particularly in the upper range of distribution (hematocrit value >50–55%). The preoperative hematocrit histogram showed an approximately unimodal distribution, with good agreement between the theoretical normal curve and the central data, but with deviations at the extremes (Figure 7a,b). The Q–Q plot similarly showed that the most observations aligned closely with the theoretical diagonal, although notable departures were present at both ends of the distribution, especially among higher values. These results confirm the influence of extreme observations on the overall normality of the data and support the subsequent use of nonparametric statistical methods.

**Figure 8:**
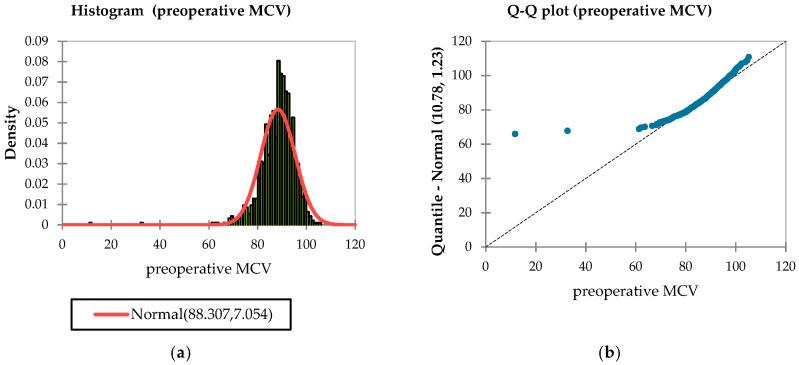
Histogram of preoperative MCV values in the study cohort (**a**), with the superimposed theoretical normal distribution curve, and the corresponding Q–Q plot (**b**).

The mean corpuscular volume (MCV) values ranged widely from 11.7 fL to 105.2 fL, reflecting substantial heterogeneity in hematologic status within the analyzed cohort. The mean MCV was 88.3 fL ± (7.05 fL), with a closely corresponding median of 89.0 fL (IQR 84.7–92.7 fL) suggesting a distribution centered around values consistent with normocytic indices. The moderate dispersion of the values indicates that most patients had MCV measurements within a physiologic or only mildly altered range for pregnancy. However, analysis of distributional shape revealed significant departures from normality. Marked negative skewness (Pearson’s skewness = −2.33) indicates an extended left tail toward lower MCV values, suggesting the presence of a subgroup of patients with pronounced microcytosis (Figure 8a,b).

### 3.2. Hemoglobin Dynamics and Transfusion Management: Univariate Analysis Using the Chi-Square Test

To assess the association between the severity of anaemia at admission and the need for postoperative transfusion support, the Pearson chi-square test was applied. The results demonstrated a highly statistically significant correlation between the two variables (χ^2^ = 99.56, df = 4, *p* < 0.001) (Table 2). These findings support the study hypothesis that optimization of hemoglobin levels through preoperative treatment significantly reduces the incidence of postoperative red blood cell transfusions.

**Figure 9:**
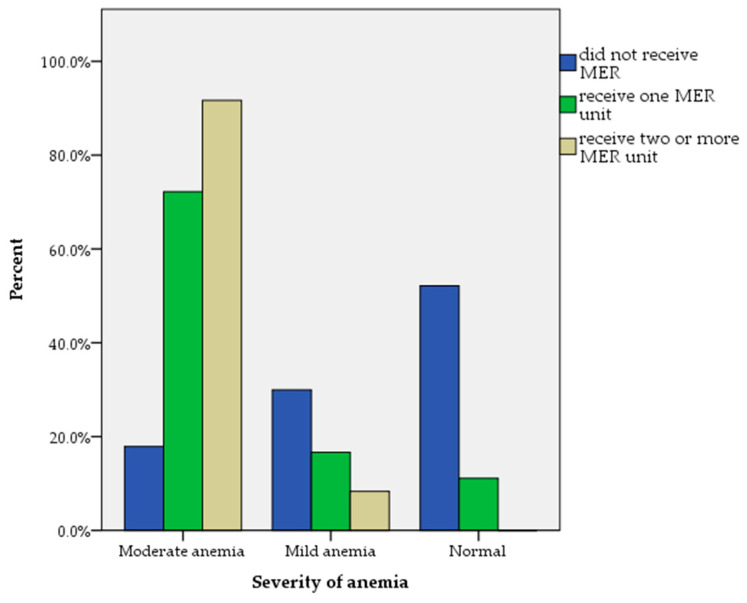
Distribution of blood transfusion requirements according to the severity of anaemia.

Figure 9 illustrates the distribution of red blood cell transfusion requirements according to the severity of preoperative anaemia. The indication for transfusion was standardized across the study cohort, primarily triggered by a hemoglobin threshold below 7 g/dL, following blood loss during or after delivery. This clinical approach ensured that transfusions were administered based on both objective laboratory values and hemodynamic stability. A clear trend is observed: patients classified as having normal hemoglobin levels required for transfusion at negligible rates. By contrast, as anaemia progressed to moderate severity, the incidence of packed red blood cell administration increased, including both single-unit and multiple-unit transfusions. The absence of cases of severe preoperative anaemia in the study cohort reflects the effectiveness of antenatal screening and first-line management, with patients being optimized before the time of delivery.

This correlation underscores the fact that preoperative anaemia therapy is not merely a measure of biological optimization, but a fundamental patient blood management strategy capable of substantially reducing dependence on allogeneic blood products and the risks associated with their use.

### 3.3. Influence of Anticoagulant Therapy and Mode of Delivery on Transfusion Risk: A Logistic Regression Analysis

To assess the predictive power of the analyzed factors on transfusion requirements, we applied a multiple logistic regression model. The results (Table 3) show that preoperative hemoglobin level is the main independent predictor of red blood cell transfusion (*p* < 0.001). The odds ratio (OR = 0.216) indicates a marked clinical effect: each 1 g/dL increase in admission hemoglobin reduces the probability of transfusion by 78.4% (95% CI: 0.153–0.306). Notably, the model suggests that when hemoglobin is optimized, other factors such as age (*p* = 0.74) or anticoagulant therapy (*p* = 0.40) lose their significance as independent predictors.

These results reinforce the central hypothesis of the study, suggesting that active management of preoperative anaemia represents the most influential factor in reducing transfusion requirements, surpassing the contribution of demographic characteristics and associated comorbidities.

### 3.4. Predictive Performance of Preoperative Hemoglobin: ROC Curve

**Figure 10:**
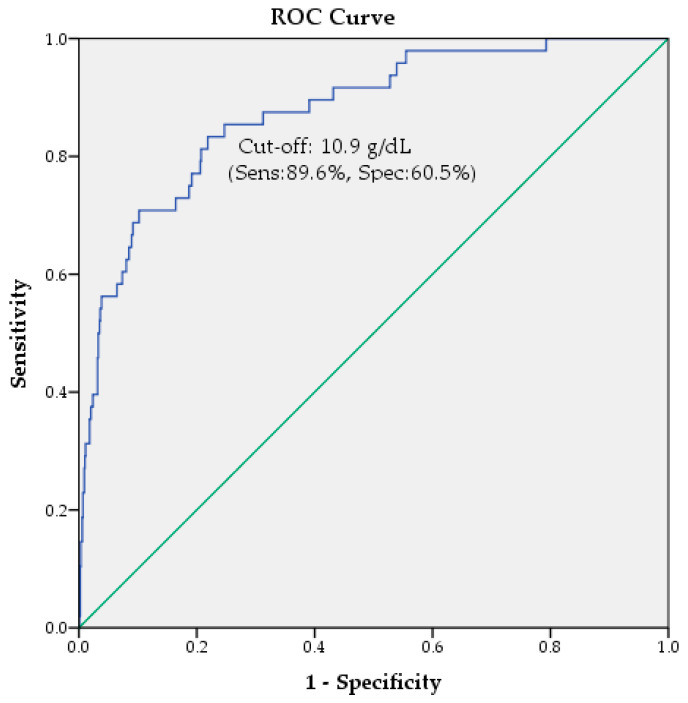
Multivariate ROC curve analysis incorporating preoperative hemoglobin and BSA as predictors of packed red blood cell transfusion requirement. The diagonal green line represents the reference line for a non-discriminatory test (AUC = 0.5).

The predictive performance of preoperative hemoglobin and BSA for transfusion requirements was evaluated by ROC curve analysis (Figure 10). The area under the curve (AUC) was 0.875 (95% CI: 0.823–0.92, *p* < 0.001), indicating high predictive accuracy. These findings suggest that monitoring and optimizing preoperative hemoglobin represent the most effective screening strategy for identifying patients at increased risk of hemorrhage, surpassing other clinical factors such as age or anticoagulant therapy.

The analysis of the ROC curve coordinates identified the optimal cut-off for preoperative hemoglobin in predicting transfusion requirement. Using the Youden index (J), this threshold yielded a sensitivity of 89.6% and a specificity of 60.5%, with a maximum J value of 0.592.

Clinically, these findings indicate that patients admitted with hemoglobin values below 10.9 g/dL carry a substantially increased likelihood of requiring perioperative transfusion support, underscoring this threshold as a critical trigger for implementation of PBM strategies.

## 4. Discussion

The core finding of this study is the identification of a preoperative Hb threshold of 10.9 g/dL as a critical predictor for red blood cell transfusion. Our ROC curve analysis yielded an AUC of 0.875, demonstrating an ‘excellent’ discriminatory power according to standard diagnostic scales. Clinically, a cut-off of 10.9 g/dL provides a strategic balance between sensitivity (89.6%) and specificity (60.5%), effectively capturing the vast majority of patients at risk for transfusion while minimizing unnecessary clinical alerts for those with adequate hematological reserves. This value is remarkably consistent with the WHO definition of anemia in the third trimester (Hb < 11.0 g/dL), reinforcing the biological plausibility of our model. In the context of PBM, this threshold should not merely be viewed as a laboratory marker, but as a ‘clinical trigger’ for immediate intervention. Patients approaching delivery with a hemoglobin level below this point possess a significantly reduced physiological buffer to withstand the inherent blood loss of childbirth, leading to a precipitous transition toward allogeneic transfusion to maintain tissue oxygenation.

Given the large sample size (n = 932), the formal tests of normality (Shapiro–Wilk and Anderson–Darling) frequently showed statistically significant departures from normality (*p* < 0.05), including for variables whose distributions were close to normal. Age, height, preoperative hemoglobin, and postoperative hemoglobin exhibited approximately symmetric distributions, with skewness and kurtosis values close to zero and good alignment on Q–Q plots, and were therefore considered practically normally distributed. These variables were summarized as mean ± standard deviation and could subsequently be analyzed using parametric tests, provided that the other statistical assumptions were also satisfied. By contrast, body weight, preoperative platelet count, hematocrit, and MCV showed asymmetric distributions, with higher skewness and/or kurtosis values and marked departures on Q–Q plots, suggesting the influence of extreme values and the biological heterogeneity characteristic of the pregnant patient cohort.

Considering the characteristics of the study cohort, the clinical interpretation of hemoglobin values should be made in accordance with thresholds specific to pregnancy. According to international guidelines, anaemia in pregnancy is defined as hemoglobin levels below 11 g/dL, regardless of gestational age. Anaemia may be classified as follows:

• Mild anaemia: 10.0–10.9 g/dL;
• Moderate anaemia: 7.0–9.9 g/dL;
• Severe anaemia: < 7.0 g/dL.

Within this framework, the mean preoperative hemoglobin value observed in our cohort (11.88 g/dL) is only slightly above the lower limit of the normal range for pregnancy, indicating that a considerable proportion of patients fell within the range associated with mild anaemia. The presence of minimum values as low as 8.0 g/dL indicates a subgroup of patients with moderate anaemia, with potential clinical implications for perioperative risk, transfusion requirements, and materno-fetal outcomes.

In this study cohort, most patients fell within the physiological range; however, a clinically relevant subgroup had platelet counts below 150 × 10^3^/µL, consistent with gestational thrombocytopenia, a common condition in pregnancy with important implications for obstetrics management.

In pregnant women, hematocrit values are influenced by the physiological hemodilution of pregnancy, resulting from a disproportionate increase in plasma volume relative to red cell mass. Accordingly, normal hematocrit values in pregnancy are generally lower, with accepted ranges of approximately 32–42%, depending on the trimester. The mean value observed in this cohort (36.5%) lies within the expected physiological range for pregnancy. Nevertheless, values below 33–34% may suggest gestational anaemia, whereas isolated elevated values may reflect hemoconcentration.

These findings support the implementation of a rigorous protocol for the detection and treatment of preoperative anaemia. Therapeutic strategies should be individualized according to the risk profile: it has been suggested in the literature that patients receiving anticoagulant therapy may benefit from a higher Hb reserve, potentially above 12 g/dL, to mitigate hemorrhage risk. Similarly, patients scheduled for caesarean section should be monitored more closely; given the statistically demonstrated risk of a marked decline in hemoglobin, the correction of moderate anaemia represents the intervention with the most favorable cost–benefit ratio for reducing transfusion rates. Any patient with Hb below 10.9 g/dL could be considered at a high risk for transfusion and may require individualized management.

The findings of this study extend the current PBM-oriented literature, which highlights the importance of identifying and treating antenatal anaemia but rarely defines explicit, data-derived hemoglobin thresholds to trigger intervention. By quantifying the effect size of preoperative hemoglobin through multivariate modelling and by proposing a ROC-based cut-off with high discriminative performance, the present analysis provides a concrete, clinically applicable instrument that can translate the PBM principles into routine obstetrics practice and help narrow the gap between broad policy recommendations and individualized clinical decision-making [23].

The present analysis demonstrates that preoperative hemoglobin is the principal independent determinant of red blood cell transfusion in the obstetrics population studied, with each 1 g/dL increment at admission conferring an almost 80% reduction in transfusion odds and an associated optimal predictive threshold of 10.9 g/dL. This pattern is in close agreement with data from large tertiary-care cohorts, where antenatal anaemia has been linked to a several-fold increase in transfusion risk and a graded, inverse association between predelivery hemoglobin and the likelihood of packed red cell administration. In those settings, an optimal hemoglobin threshold of roughly 10.6 g/dL has been reported, with high sensitivity and specificity for predicting transfusion, a value that closely approximates the 10.9 g/dL cut-off identified in our sample. The concordance between these estimates suggests that the hemoglobin inflection point for transfusion risk is relatively stable across different clinical environments and supports the use of preoperative hemoglobin as a practical trigger for Patient Blood Management interventions, rather than as a merely descriptive laboratory metric [24].

This study possesses several notable strengths, primarily the large and diverse sample size of 932 pregnant women, which enhances the statistical power of our predictive model. By applying rigorous exclusion criteria—specifically removing patients with chronic hematological conditions such as Thalassemia Major or non-iron deficiency anemias—we ensured that the observed outcomes directly reflect the impact of gestational iron deficiency on transfusion risk. Furthermore, the use of ROC curve analysis allowed for the identification of a precise, clinically actionable Hb threshold (10.9 g/dL), providing a data-driven basis for Patient Blood Management (PBM) protocols. However, certain limitations must be acknowledged. The retrospective, single-center design may limit the generalizability of the findings to different geographical regions or healthcare settings with varying transfusion triggers. Additionally, while we controlled for major confounders such as anticoagulant therapy and delivery mode, other factors—including intraoperative surgical complexity or specific postpartum hemorrhage (PPH) management protocols—were not fully captured in the electronic records. Despite these constraints, the high AUC and the significant odds ratios obtained underscore the robustness of preoperative hemoglobin as a critical independent predictor of transfusion requirements in the obstetrics population.

Our findings suggest that 10.9 g/dL may serve as a critical trigger for PBM protocols. The clinical consideration of initiating intravenous iron therapy in pregnant patients who reach this threshold during the third trimester merits further investigation, as it could potentially reduce the rate of allogeneic transfusions and the associated costs.

Our study has several key strengths. First, it utilizes a robust PBM (Patient Blood Management) framework tailored to a specific and vulnerable population—pregnant women—addressing a critical gap in obstetric care. The use of rigorous statistical methods, including logistic regression and ROC analysis, provides a high level of predictive accuracy (AUC = 0.875), which enhances the reliability of our findings for clinical decision-making regarding transfusion needs.

However, some limitations must be acknowledged. This was a single-center and retrospective study, which may limit the generalizability of the results to other clinical settings or diverse ethnic populations. Additionally, while we focused on hemoglobin levels and mode of delivery, other potential confounders such as nutritional status or specific co-morbidities were not fully accounted for. Future prospective, multi-center studies are needed to validate these predictive models across broader populations.

## 5. Conclusions

Our observational study indicates that preoperative hemoglobin acts as a significant independent predictor of red blood cell transfusion requirements in the obstetric population, showing higher predictive value compared to age or anticoagulant therapy. The established threshold of 10.9 g/dL demonstrated an AUC of 0.875; however, we acknowledge that the model relied on a limited set of variables. Future refinements, such as replacing height and weight with BSA, could potentially enhance the model’s predictive accuracy beyond the current AUC. These findings suggest the benefit of a proactive PBM-oriented approach, specifically focusing on the first pillar: the optimization of red cell mass prior to delivery. We conclude that implementing targeted screening and rapid correction of iron deficiency for any pregnant woman falling below this 10.9 g/dL mark—especially in the third trimester—is essential to enhance maternal safety, preserve blood bank resources, and reduce the morbidity associated with allogeneic transfusions. Future protocols should integrate this data-driven threshold to standardize hematological optimization in both elective and emergency obstetric settings.

## Figures and Tables

**Table 1:** Mean and median values of the continuous variables.

Variable	Minimum	Maximum	Median	Mean
Age	18.000	40.000	28 (23–33)	28.03 ± 5.71
Weight	41.600	138.600	77 (69–87.5)	78.91 ± 14.66
Height	140.000	190.000	164 (160–168)	163.55 ± 6.95
Preoperative hemoglobin	8.000	15.300	11.9 (11.1–12.7)	11.88 ± 1.18
Preoperative platelet count ×10^3^	106.000	495.000	244 (200–294)	251.57 ± 69.25
Preoperative hematocrit	23.700	72.600	36.6 (34.3–38.8)	36.53 ± 3.57
Preoperative MCV ^1^	11.700	105.200	89 (84.6–92.7)	88.31 ± 7.05
Postoperative hemoglobin	6.700	14.200	10.8 (10–11.7)	10.78 ± 1.23

^1^ MCV—Mean Corpuscular Volume.

**Table 2:** Chi-Square Tests.

	Value	df	Asymp. Sig. (2-Sided)
Pearson Chi-Square	99.566 ^a^	4	0.000
Likelihood Ratio	80.892	4	0.000
Linear-by-Linear Association	75.238	1	0.000
N of Valid Cases	932		

^a^ Two cells (22.2%) have an expected count less than 5. The minimum expected count is 2.51.

**Table 3:** Binary logistic regression.

Variables Step 1 ^a^	B	S.E.	Wald	df	Sig.	Exp(B)	95% C.I. for Exp(B)	
							Lower	Upper
Hb level	−1.532	0.177	74.600	1	0.000	0.216	0.153	0.306
Anticoagulant therapy	−0.410	0.494	0.688	1	0.407	0.663	0.252	1.749
			8.798	2	0.012			
Mode of delivery (1)	17.597	40,193.353	0.000	1	1.000	43,894,118.406	0.000	
Mode of delivery (2)	18.852	40,193.353	0.000	1	1.000	153,903,876.154	0.000	
age	−0.010	0.030	0.110	1	0.740	0.990	0.934	1.050
constant	−3.884	40,193.353	0.000	1	1.000	0.021		

^a^ Variables entered on step 1: hemoglobin level, anticoagulant therapy, mode of delivery, age.

## Data Availability

The database used in this study can be found in the hospital informatic system and archives and is not available for public access.

## Abbreviations

The following abbreviations are used in this manuscript:

PBM	Patient Blood Management
Hb	Hemoglobin
ROC	Receiver Operating Characteristic
AUC	Area Under the Curve
WHO	World Health Organization
MER	Erythrocyte Mass
IDA	Iron Deficiency Anemia
